# The influence of prostatic *Cutibacterium acnes* infection on serum levels of IL6 and CXCL8 in prostate cancer patients

**DOI:** 10.1186/s13027-018-0204-7

**Published:** 2018-11-14

**Authors:** Henrik Ugge, Jessica Carlsson, Bo Söderquist, Katja Fall, Ove Andén, Sabina Davidsson

**Affiliations:** 1 Department of Urology, Faculty of Medicine and Health, Örebro University, 701 85 Örebro, Sweden; 2 Faculty of Medicine and Health, Örebro University, Örebro, Sweden; 3 Clinical Epidemiology and Biostatistics, School of Medical Sciences, Örebro University, Örebro, Sweden; 4 Department of Medical Epidemiology, Karolinska Institutet, Stockholm, Sweden

**Keywords:** Prostate cancer, Inflammation, *Cutibacterium acnes*, IL6, CXCL8, Cytokines

## Abstract

**Background:**

Chronic prostatic inflammation, caused by *Cutibacterium acnes (C. acnes),* has been proposed to influence the risk of prostate cancer development. In vitro studies have demonstrated the capacity of *C. acnes* to induce secretion of Interleukin 6 (IL6) and C-X-C motif chemokine ligand 8 (CXCL8) by prostate epithelial cells. Both these inflammatory mediators have been implicated in prostate cancer pathophysiology. In this cohort study, we aimed to investigate the influence of prostatic *C. acnes* on serum levels of IL6 and CXCL8.

**Methods:**

We recruited 99 prostate cancer patients who underwent radical prostatectomy at Örebro University Hospital. The cultivation of pre-operatively obtained prostate biopsies identified *C. acnes* in 60 of the 99 patients. Levels of IL6 and CXCL8 in pre-operative serum samples were analyzed using ELISA, and concentrations were compared between prostate cancer patients with and without prostatic *C. acnes* infection using standard statistical methods.

**Results:**

No statistical differences were observed in serum levels of IL6 and CXCL8 between subjects with and without prostatic *C. acnes* infection.

**Conclusions:**

Our results indicate that prostatic *C. acnes* infection may give rise to low-grade inflammation with little effect on systemic levels of IL6 and CXCL8.

## Introduction

Inflammation is recognized as a critical component of tumor progression and may be involved in the development of several types of cancer [1]. It has further been hypothesized to influence both prostate cancer development and progression [2]. Both acute and chronic inflammation are frequently observed in prostate cancer specimens obtained from biopsy sampling, transurethral prostate resection and prostatectomies [3–5].

The inflammatory microenvironment is believed to contribute to the development of prostate cancer via a number of mechanisms, including production of reactive molecule species with known capacity to cause DNA damage [1]. In addition, increased levels of specific cytokines and chemokines in a persistently inflamed tissue have been suggested to contribute to prostate cancer initiation, promotion, and progression [2, 6]. Two of the most frequently studied cytokines and chemokines in relation to prostate cancer development and pathophysiology are Interleukin-6 (IL6) and C-X-C motif chemokine ligand 8 (CXCL8). Several lines of evidence have suggested associations between IL6 and CXCL8, and prostate cancer aggressiveness, progression, and poor clinical outcome [2, 6–11].

The commonly observed prostatic inflammation may be caused by different etiological factors, including infectious agents such as bacteria. *Cutibacterium acnes (C. acnes),* formerly *Propionibacterium acnes*, is an anaerobic Gram-positive rod-shaped bacterium, generally regarded as commensal of the skin, but also commonly associated with the skin condition acne vulgaris [12]. *C. acnes* has further been identified as the etiological agent in a number of low-grade infections; predominantly implant-associated infections, related to for example prosthetic joint devices, prosthetic heart valves and neurosurgical shunts [13].

An impact of *C. acnes* in prostate pathologies has been suggested, since several independent studies have observed it to be the most prevalent microorganism in prostate tissue [14–16]. Cohen et al. detected *C. acnes* in 35% of cultures from prostatectomy samples, and observed a significantly higher degree of inflammation in samples positive for *C. acnes* compared to negative samples [17]. These findings raised the question whether *C. acnes* could be involved in inflammation-induced prostate cancer development. Results from recent studies have supported the theory that *C. acnes* may influence prostate cancer development by causing a chronic inflammation of the prostate [16, 18–21]. In line with the suggested role of *C. acnes* in prostate cancer development, our group reported the bacterium to be significantly more common in prostate tissue obtained from men with prostate cancer compared to men without the disease [16].

Additional findings supporting the hypothesis of *C. acnes* as a trigger of prostate cancer development comes from in vitro studies reporting an increased secretion of IL6 and CXCL8 by prostate epithelial cells infected with *C. acnes* [16, 21, 22]. Alexeyev et al. further detected *C. acnes* in prostate samples from individual patients taken up to six years apart, indicating a chronic *C. acnes* infection [23]. This is possibly facilitated by a capacity of *C. acnes* to evade eradication by immune cells [24–26]. A low-grade chronic inflammation caused by prostatic *C. acnes* infection, including induced secretion of IL6 and CXCL8, may thus over time contribute to prostate cancer development.

In order to evaluate whether prostatic *C. acnes* infection activates a systemic inflammatory response, or rather contributes to a low-grade localized inflammation of the prostate, the aim of the present study was to investigate whether prostatic *C. acnes* infections influence serum levels of IL6 and CXCL8 in prostate cancer patients.

## Material and methods

### Study population

A total number of 99 consecutive patients undergoing radical prostatectomy at Örebro University Hospital between 2009 and 2012 were included in this study. The techniques for biopsy and *C. acnes* characterization have been described in detail previously [16]. Prostate biopsies were obtained under sterile conditions at the operating theatre, after extraction of prostatectomy specimens. Culture and species verification of *C. acnes* were performed using routine microbiological procedures. *C. acnes* was isolated in samples from 61% of the patients (*n* = 60). Baseline characteristics were obtained from patient files and pathology reports. The study was approved by the regional ethical review board, Uppsala, Sweden (reference 2008/293).

### ELISA-analysis

In order to investigate the serum levels of IL6 and CXCL8 in men with and without prostatic *C. acnes* infection, we collected 10 ml blood from each patient prior to surgery. Blood samples were allowed to clot for 30 min at room temperature (RT) and serum was obtained by centrifugation at 3000 *x* g for 10 min at RT. Serum samples were then aliquoted and stored at − 80 ° C pending analysis. All samples were subsequently analyzed retrospectively, and the individual performing measurements was blinded to *C. acnes* infection status of the respective study participants.

Serum samples were thawed on ice, and concentrations of IL6 and CXCL8 were measured using commercially available ELISA kits (ELISA Quantikine HS, catalog numbers HS600B (IL6) and HS800 (CXCL8), R&D systems, Minneapolis, USA), according to manufacturer’s instructions. The minimum detectable levels were 0.11 pg/ml for IL6 and 0.4 pg/ml for CXCL8, and the detection ranges were 0.2–10 pg/ml and 1–64 pg/ml respectively. The optical density was measured using a Multiskan Ascent plate reader (Thermo labsystems, Helsinki, Finland) at 490 nm and 620 nm. The reference absorbance at 620 nm was subtracted from 450 nm in order to correct for background noise.

All samples were measured in duplicates, and samples where re-measured if one of the replicates failed to be measured. Blanks and standards were assayed according to the manufacturer’s instructions. The mean values of absorbance vs. concentrations were plotted and a 4-parameter logistic (4PL) non-linear regression model fit was applied, and R^2^-values above 0.9 were considered as acceptable.

### Statistical analysis

Mean replicate serum concentrations of IL6 and CXCL8 in each sample were used in the statistical analyses. Descriptive statistics included frequencies, proportions, medians and ranges. We used QQ-plots, histograms, box plots and Shapiro-Wilk Tests to evaluate continuous variables for normality. Prostate specific antigen (PSA), age at surgery, prostate volume (ultrasound-estimated), IL6, and CXCL8 were all found to be non-normally distributed. Differences between groups were analyzed using Chi-square Test, Mann-Whitney U Test or Kruskal-Wallis Test, where applicable. Associations between continuous variables were evaluated using non-parametric correlation, as well as by categorizing the exposure variable into quartiles and comparing medians of outcome variable.

To test for confounding by age or other factors of the association between prostatic C. acnes infection and serum levels of CXCL8 and IL6 we performed simple and multiple linear regression analyses with CXCL8 and IL6 as continuous outcome variables. Outcome variables were transformed using the natural logarithm in order to normalize distribution. Outlying observations of > 3 inter-quartile ranges (IQRs) were further excluded (*n* = 4 for CXCL8, *n* = 0 for IL6). Assumptions of normality, homoscedasticity and linear relationship between exposure and outcome were assessed visually using histogram of residual distributions, P-P plots, and plots of residuals vs. predicted values and exposure variables respectively. Influence diagnostics were performed using Cook’s distance and multicollinearity was assessed using variance inflation factors (VIF). The simple linear regression models included *C. acnes* status as exposure and CXCL8/IL6 respectively as outcome. The multiple linear regression included Gleason sum (categorical; 6, 3 + 4, 4 + 3, 8+), age (continuous, years), PSA (continuous, ng/ml, transformed using the natural logarithm) and prostate volume (ml, continuous). We also tried a multiple linear regression model with continuous variables transformed into categorical variables, by dividing into quartiles. Covariates included in model were selected based on biological plausibility. All analyses were performed using IBM SPSS Statistics version 22 (IBM Corp. Armonk, NY, USA), and statistical significance was defined as *p* < 0.05.

Based on previous observations of serum levels and standard deviations (SD) of IL6 and CXCL8 in healthy individuals [27, 28], the population size of the current study, and assuming a β of 0.2, the study is powered to detect an effect size of 0.39 SD. This corresponds to at least a medium effect size (> 0.5 SD) as defined by Cohen [29].

## Results

### Baseline characteristics

A total number of 99 patients were eligible for analyses. The median age of the cohort was 64.6 years (range: 53.8–71.6 years), and the median preoperative PSA was 6.3 ng/ml (range: 2.1–43.0 ng/ml). The Gleason score (GS) distribution of the patients was: GS 6: *n* = 36, GS 7: *n* = 59 (GS 3 + 4: *n* = 43, GS 4 + 3: *n* = 16), GS 8: *n* = 2, GS 9: n = 2, and GS 10: *n* = 0. The pathologic stages were pT2 (*n* = 88) and pT3 (*n* = 11), and 34 patients had positive surgical margins. Men with prostatic *C. acnes* infection were on average slightly older than those without *C. acnes* infection (median age 65.1 vs. 63.1 years, *p* = 0.047). Gleason score, PSA, pT-stage, ultra-sound estimation of prostate volume, and frequency of positive surgical margins did not differ between patients positive or negative for prostatic *C. acnes* infection (Table 1).

**Table 1:** Chararacteristics of study participants (*n* = 99), categorized by negative or positive finding of *C. acnes*

	*C. acnes +* (*n* = 60)	*C. acnes-* (*n* = 39)	*p*-value
Age at time of surgery, years			
Median (min-max)	65.1 (54.0–71.0)	63.1 (53.8–71.6)	*p* = 0.047^a^
PSA before surgery, ng/ml			
Median (min-max)	6.4 (2.1–43)	5.9 (3.2–28)	*p* = 0.450^a^
Prostate volume, ml			
Median (min-max)	32 (10–138)	35 (17–115)	*p* = 0.314^a^
Gleason score from prostatectomy sample, n (%)			
Gleason 6	25 (41.7%)	11 (28.2%)	*p* = 0.170^b^
Gleason 3 + 4 = 7	25 (41.7%)	18 (46.2%)	
Gleason 4 + 3 = 7	8 (13.3%)	8 (20.5%)	
Gleason 8	2 (3.3%)	0 (0%)	
Gleason 9–10	0 (0%)	2 (5.1%)	
Positive surgical margins n (%)	22 (36.7%)	12 (30.8%)	*p* = 0.546^b^
pT-stage, n (%)			
pT2a	8 (13.3%)	5 (12.8%)	*p* = 0.149^b^
pT2c	46 (76.7%)	29 (74.4%)	
pT3a	6 (10.0%)	2 (5.1%)	
pT3b	0 (0%)	3 (7.7%)	

^a^Mann-Whitney U test

^b^Chi-square test

### Serum levels of IL6 and CXCL8 in relation to various patient characteristics

Due to insufficient amount of serum, we were unable to measure concentrations of IL6 in one sample and CXCL8 in four additional samples. For patients with available cytokine data, the median pre-operative serum levels of IL6 and CXCL8 were 1.09 pg/ml (range 0.30–3.76 pg/ml) and 9.97 pg/ml (range 3.36–155.53 pg/ml), respectively. Median serum levels of IL6 and CXCL8 did not differ when comparing groups based on GS, pT-stage, surgical margins, PSA, age and prostate volume (Table 2 and Table 3). Non-parametric correlation did further not indicate any significant correlation between IL6 or CXCL8 and age, PSA or prostate volume respectively (Table 2 and Table 3).

**Table 2:** Preoperative serum levels of IL6 (pg/ml) in relation to other variables for men who underwent radical prostatectomy and had available information on serum IL6 (*n* = 98)

Total population (*n* = 98)					
Median serum IL6, pg/ml (min-max)	1.09 (0.30–3.76)				
By *C. acnes* category	*C. acnes +* (*n* = 60)		*C. acnes-* (*n* = 38)		
Median serum IL6, pg/ml (min-max)	1.04 (0.35–3.76)		1.16 (0.30–3.13)		*p* = 0.106^a^
Gleason sum	Gleason 6 (*n* = 36)	Gleason 3 + 4 (*n* = 42)	Gleason 4 + 3 (*n* = 16)	Gleason 8+ (*n* = 4)	
Median serum IL6, pg/ml (min-max)	1.02 (0.3–3.76)	1.09 (0.45–3.13)	1.14 (0.47–2.05)	1.12 (0.7–1.37)	*p* = 0.836^a^
Positive sugical margins	No (*n* = 64)		Yes (*n* = 34)		
Median serum IL6, pg/ml (min-max)	1.12 (0.3–3.76)		1.03 (0.39–2.97)		*p* = 0.420^a^
pT stage	pT2 (*n* = 87)		pT3 (*n* = 11)		
Median serum IL6, pg/ml (min-max)	1.1 (0.3–3.76)		1.05 (0.61–2.06)		*p* = 0.862^a^
PSA					
*By PSA quartile*	< 4.6 ng/ml	≥4.6, < 6.4 ng/ml	≥6.4, < 9.0 ng/ml	≥9 ng/ml	
Median serum IL6, pg/ml (min-max)	1.02 (0.45–2.88)	1.08 (0.47–2.23)	1.12 (0.3–3.13)	1.16 (0.39–3.76)	*p* = 0.601^a^
*Non-parametric correlation*					
Correlation coefficient	0.058				*p* = 0.571^b^
Age					
*By age quartile*	< 61.3 years	≥61.3, < 64.7 years	≥64.7, < 67.1 years	≥67.1 years	
Median serum IL6, pg/ml (min-max)	1.09 (0.3–3.13)	0.93 (0.39–2.97)	1.17 (0.47–3.76)	1.11 (0.35–2.88)	*p* = 0.416^a^
*Non-parametric correlation*					
Correlation coefficient	0.113				*p* = 0.270^b^
Prostate volume					
*By volume quartile*	10–26 ml	27–33 ml	34–50 ml	51–138 ml	
Median serum IL6, pg/ml (min-max)	1.39 (0.3–3.76)	1.1 (0.56–2.68)	1.06 (0.39–2.73)	1.1 (0.35–2.78)	*p* = 0.397^a^
*Non-parametric correlation*					
Correlation coefficient	−0.106				*p* = 0.321^b^

^a^*p*-value fron Kruskal-Wallis Test/Mann-Whitney U Test; ^b^*p*-value from Spearman correllation

**Table 3:** Preoperative serum levels of CXCL8 (pg/ml) in relation to other variables for men who underwent radical prostatectomy and had available information on serum CXCL8 (*n* = 95)

Total population (n = 95)					
Median serum CXCL8, pg/ml (min-max)	9.97 (3.36–155.53)				
By *C. acnes* category	*C. acnes +* (*n* = 58)		*C. acnes-* (*n* = 37)		
Median serum CXCL8, pg/ml (min-max)	10.01 (5.05–130.93)		9.68 (3.36–155.53)		*p* = 0.658^a^
Gleason sum	Gleason 6 (*n* = 36)	Gleason 3 + 4 (*n* = 41)	Gleason 4 + 3 (*n* = 15)	Gleason 8+ (*n* = 3)	
Median serum CXCL8, pg/ml (min-max)	10.05 (4–155.53)	9.21 (3.36–83.2)	9.96 (5.95–16.07)	11.16 (9.41–12.14)	*p* = 0.843^a^
Positive sugical margins	No (*n* = 62)		Yes (*n* = 33)		
Median serum CXCL8, pg/ml (min-max)	10.01 (3.36–155.53)		9.41 (4.79–38.16)		*p* = 0.344^a^
pT stage	pT2 (*n* = 86)		pT3 (n = 9)		
Median serum CXCL8, pg/ml (min-max)	9.99 (3.36–155.53)		9.41 (6.24–20.53)		*p* = 0.576^a^
PSA					
*By PSA quartile*	< 4.6 ng/ml	≥4.6, < 6.4 ng/ml	≥6.4, < 9.0 ng/ml	≥9 ng/ml	
Median serum CXCL8, pg/ml (min-max)	9,68 (5,05-130,93)	10,03 (4,79-155,53)	8,93 (4–38,16)	10,65 (3,36-83,2)	*p* = 0.646^a^
*Non-parametric correlation*					
Correlation coefficient	0.069				*p* = 0.505^b^
Age					
*By age quartile*	< 61.3 years	≥61.3, < 64.7 years	≥64.7, < 67.1 years	≥67.1 years	
Median serum CXCL8, pg/ml (min-max)	10,09 (3,36-155,53)	10,09 (3,36-155,53)	9,21 (5,26-83,2)	11,16 (5,4-62,26)	
*Non-parametric correlation*					
Correlation coefficient	0.044				*p* = 0.674^b^
Prostate volume					
*By volume quartile*	0–26 ml	27–33 ml	34–50 ml	51–138 ml	
Median serum CXCL8, pg/ml (min-max)	10.28 (6.24–16.68)	8.89 (3.36–83.2)	10.8 (4–130.93)	9.68 (5.26–155.53)	*p* = 0.468^a^
*Non-parametric correlation*					
Correlation coefficient	0.045				*p* = 0.681^b^

^a^*p*-value fron Kruskal-Wallis Test/Mann-Whitney U Test; ^b^*p*-value from Spearman correllation

### Serum levels of IL6 and CXCL8 in men with and without prostatic C. acnes infection

Median levels of IL6 for patients with and without prostatic *C. acnes* infection were 1.04 pg/ml and 1.16 pg/ml, respectively (Table 2). For CXCL8, the corresponding values were 10.01 pg/ml and 9.68 pg/ml (Table 3). Comparison of patients with and without prostatic *C. acnes* infection did not indicate statistically significant differences in serum levels of IL6 or CXCL8.

Simple linear regression did not indicate significant association between *C. acnes* status and IL6 (log-transformed); regerssion coefficient (β): -0.17; 95% Confidence interval (95% CI): -0.39, 0.06; *p* = 0.143. Adjustment for multiple covariates did not change coefficients markedly (β: -0.17, 95% CI: -0.43, 0.09, *p* = 0.187). The regression coefficient for simple linear regression of *C. acnes* on CXCL8 (log-transformed) was 0.11 (95% CI -0.07, 0.30, *p* = 0,234) and for the multivariable model 0.09 (95% CI -0.11, 029, *p* = 0,361), likewise suggesting absence of association. Exclusion of individual covariates, and categorization of model covariates did not bring about significant changes in parameter estimates (data not shown). Regression diagnostics did not indicate violation of assumptions after transformation of outcome variables and exclusion of outliers in CXCL8. In summary, we did not find evidence for confounding of the association between *C. acnes* and serum levels of CXCL8 and IL6, and no further evidence of a significant association.

## Discussion

Previous studies have shown that *C. acnes* has the capability to stimulate secretion of IL6 and CXCL8 by prostate epithelial cells in vitro [16, 21, 22], and a microenvironment containing high levels of these two inflammatory mediators has been associated with prostate cancer progression [6–8, 10, 11]. In the present study, we aimed to evaluate the influence of prostatic *C. acnes* infection on systemic levels of IL6 and CXCL8 in prostate cancer patients. When comparing patients with and without prostatic *C. acnes* infection, no significant differences were observed in serum levels of either mediator.

Chronic inflammation is considered involved in the development of several types of cancer [1], and multiple lines of evidence indicate that inflammation may influence prostate cancer development and progression [2]. Histological signs of prostatic inflammation are frequently observed [3, 4], and the term proliferative inflammatory atrophy (PIA) has been coined for areas characterized by atrophy, inflammation and epithelial proliferation [30]. Morphologic studies have indicated transition from PIA to high-grade prostatic intraepithelial neoplasia (HGPIN, an established precursor lesion to prostate cancer), and possibly also to prostate cancer [31, 32]. Epidemiological studies and genetic association studies have presented further evidence supporting a possible association between prostatic inflammation and prostate cancer development [9, 33].

Based on its prevalence in prostate specimens [14–16, 21], and on observed associations with prostatic inflammation [17, 22, 34, 35], *C. acnes* has been proposed as an etiological agent for prostatic inflammation. Observed associations between prostatic occurrence of *C. acnes* and prostate cancer have consequently led to the hypothesis that prostatic inflammation caused by *C. acnes* might influence prostate carcinogenesis [16, 18, 21]. There is further some epidemiological support for a positive association between acne vulgaris and prostate cancer risk [19, 20].

IL6 and CXCL8 are pro-inflammatory mediators with diverse roles in the innate immune system. IL6 is a pleiotropic molecule involved in regulation of acute phase inflammatory response and T-cell differentiation [36], and CXCL8 is associated with chemotaxis and activation of granulocytes [37]. In addition to their role in innate immune system functions, such as protection against bacterial infections, cytokines and chemokines have been proposed to contribute to cancer development and progression. Specifically, IL6 and CXCL8 have been extensively studied in relation to prostate cancer, and have both been proposed to play important roles in prostate cancer development and progression [6–8, 10, 11]. *C. acnes* has in several studies, including one using prostate-derived isolates from the present cohort, been observed to induce secretion of both IL6 and CXCL8 by prostate epithelial cells in vitro [16, 21, 22].

The fact that we did not detect a significant difference in serum levels of IL6 or CXCL8 in patients with and without prostatic *C. acnes* infection indicates that *C. acnes* is not capable of inducing a systemic response of IL6 and CXCL8. These results are in line with previous studies evaluating serum levels of IL6 as a diagnostic marker for *C. acnes*-related peri-prosthetic shoulder infections. The indolent nature of *C. acnes* infection has been proposed as a possible reason for the lack of systemic effects [38, 39]. Studies on acne vulgaris likewise suggest absence of systemic inflammatory response, estimated by C-reactive protein (CRP) level, even in severe cases [40]. It should however be noted that elevated levels of CRP was observed previously in an animal model for prostatic *C. acnes* infection [34].

The lack of a systemic response further conforms to evidence indicating a capacity of *C. acnes* to avoid detection and eradication by the immune system, and thereby cause a persistent infection. Resistance to killing by neutrophils and macrophages [26], evasion of phagosomal degradation and long-time intracellular survival in macrophages [23–25, 41], as well as biofilm formation [13, 23] constitute possible mechanisms by which the bacterium avoids elimination. Further, Sahdo et al. reported low Caspase-1 activity in macrophages when *C. acnes* isolates, cultivated from patients in the present cohort, were used as stimuli for peripheral leukocytes obtained from blood donors [42]. The same study also observed that commensal *C. acnes* isolates induced Caspase-1 activity to a greater degree than invasive strains.

A strength with this study is the demonstrated capacity of *C. acnes*, cultured from men included in the present cohort, to induce IL6 and CXCL8 secretion by prostate epithelial cells [16]. Additional strengths of the study include serum samples obtained contemporaneously with prostate samples for microbiological analysis, as well as a relatively large study population compared to other studies of inflammatory mediators in relation to *C. acnes* pathology [38, 39]. The study, however, still lacks power to detect small differences in serum levels of IL6 and CXCL8. Study limitations include the lack of CRP as a measure of systemic inflammation, and the use of only two inflammatory mediators. Future studies may consider including multiple pro- as well as anti-inflammatory mediators in order to better characterize the systemic inflammatory response to a *C. acnes* infection.

As previously reported, prostatic *C. acnes* infection was in this cohort associated with prostate cancer in a case-control setting, but not with GS, tumor stage, or PSA [16]. In the present study, we did not observe a difference in serum levels of IL6 or CXCL8 between patients with or without prostatic *C. acnes* infection. The results are consistent with *C. acnes* proposed capacity to colonize the prostate and induce a low-grade chronic prostatic inflammation, with little effect on systemic inflammatory response, but with possible implications for prostate cancer development. Evading inflammatory activation may facilitate *C. acnes’* persistence and long-term survival in prostatic tissue.

## Conclusions

Our results indicate that *C. acnes* infection of the prostate does not give rise to a measurable increase in systemic levels of IL6 and CXCL8. This is consistent with previous observations of *C. acnes* giving rise to chronic low-grade infections.

## Abbreviations

4PL: 4-parameter logistic
*C. acnes*: *Cutibacterium acnes*
CRP: C-reactive protein
CXCL8: C-X-C motif chemokine ligand 8
GS: Gleason score
HGPIN: High-grade prostatic intraepithelial neoplasia
IL6: Interleukin 6
PIA: Proliferative inflammatory atrophy
PSA: Prostate specific antigen
RT: Room temperature
SD: Standard deviation
